# British pain clinic practitioners' recognition and use of the bio-psychosocial pain management model for patients when physical interventions are ineffective or inappropriate: results of a qualitative study

**DOI:** 10.1186/1471-2474-11-51

**Published:** 2010-03-18

**Authors:** Geoffrey Harding, John Campbell, Suzanne Parsons, Anisur Rahman, Martin Underwood

**Affiliations:** 1 Peninsula Medical School (Primary Care), Smeall Building, St Luke's Campus, Exeter, UK; 2 Picker Institute Europe, Oxford, UK; 3 University College London, Windeyer Institute, London, UK; 4 Warwick Medical School, University of Warwick, Coventry, UK

## Abstract

**Background:**

To explore how chronic musculoskeletal pain is managed in multidisciplinary pain clinics for patients for whom physical interventions are inappropriate or ineffective.

**Methods:**

A qualitative study was undertaken using semi-structured interviews with twenty five members of the pain management team drawn from seven pain clinics and one pain management unit located across the UK.

**Results:**

All clinics reported using a multidisciplinary bio-psychosocial model. However the chronic pain management strategy actually focussed on psychological approaches in preference to physical approaches. These approaches were utilised by all practitioners irrespective of their discipline. Consideration of social elements such as access to social support networks to support patients in managing their chronic pain was conspicuously absent from the approaches used.

**Conclusion:**

Pain clinic practitioners readily embraced cognitive/behavioural based management strategies but relatively little consideration to the impact social factors played in managing chronic pain was reported. Consequently multidisciplinary pain clinics espousing a bio-psychosocial model of pain management may not be achieving their maximum potential.

## Background

Chronic pain is a complex experience influenced as much by patients' social and cultural environment, beliefs, expectations, attitudes, and the meanings they ascribe to their pain, as it is by biological factors. As such, biomedicine's historical concept of chronic pain as having an organic basis requiring a specific bio-medical intervention, has increasingly given way to treating chronic pain as a bio-psychosocial phenomenon requiring integrated, multidisciplinary treatment [1,2].

This treatment approach considers chronic pain to be caused by a dynamic interaction between patients' physical, psychological and social influences shaping their responses to the pain [3]. Increasingly, when such patients are seen in specialist pain clinics they are offered a programme of multidisciplinary rehabilitation. Though the content of each programme may differ, all include elements of cognitive/behavioural principles as well as physical approaches (exercises etc) to rehabilitate patients by assisting them to self-manage their chronic pain. Evidence indicates this approach to be important and of greater benefit than uni-disciplinary approaches [4-7].

A model integrating both psychological and physiological processes facilitates an understanding of pain and informs the bio-psychosocial approach to managing chronic pain [3,8-11]. Such an approach is endorsed by authoritative bodies [12,13]. Key to this model's effectiveness is the extent to which constituent members of the team integrate their professional practices in order to optimise patient care. In a previous paper [14] we reported on the beliefs and experiences of patients who had attended a chronic pain clinic. Among the themes expressed by these patients were diminishing faith in the ability of biomedicine to relieve their pain and a sense of learning to live with pain. Both these themes could be considered to concur with the bio-psychosocial model but it was not clear whether patients developed these beliefs because of the efforts of the pain clinic or independently of those efforts. To place these themes in a wider context we therefore turned our attention to practitioners' processes and procedures for managing patients' chronic pain within pain clinics. In this paper we have analysed the views of professionals working in pain clinics and a pain management unit to investigate their management of patients for whom interventions such as drug management and physical therapy were either inappropriate or ineffective. Although the physical and socio-psychological dimensions of chronic pain might best be addressed through a multidisciplinary management approach, little is known of the team members' beliefs and expectations of this approach for such patients. We interviewed staff from different disciplines (e.g. medical, physiotherapy, psychology, nursing) to see how their background and training might influence their interactions with patients, other staff and the treatment models that they employed.

## Method

A qualitative approach was used to capture the processes and procedures used in varying organisational clinic settings and the beliefs and expectations of multidisciplinary team members in managing chronic pain patients for whom physical interventions were either ineffective or not appropriate.

### Participants

We conducted semi-structured interviews with 25 members of the multidisciplinary team representing medicine, nursing, physiotherapy and psychology drawn from seven hospital-based pain clinics and one pain management unit*.

* Pain clinics offer various multidisciplinary treatments, including cognitive based therapies, injections and prescribing medicines and are characteristically led by doctors. Each of the pain clinics sampled offered a range of individual therapies. Pain management units offer psychologically-based rehabilitative treatment for people with chronic pain and are typically led by clinical psychologists. The pain management unit we sampled offered both group based and individual pain management programmes.

Configuration of pain clinics and composition of their teams vary across Britain and their clinical lead may be drawn from differing medical specialities. In order to ensure a broadly representative sample the clinics were purposively selected to incorporate those led by anaesthetists, rheumatologists, and combined rheumatologist/anaesthetist clinics, and were geographically distributed across four NHS regions of the UK North-East, North West, London and South West. No clinic we approached declined to participate. Included in this sample was a pain clinic from which, in our previous study we had drawn a sample of patients to explore their perspectives on pain management. Interviews lasted approximately 30 minutes and were conducted during working hours in the clinic, by appointment with practitioners between clinic commitments, and undertaken by a social scientist (GH) experienced in qualitative interviews around chronic pain issues. Interviewees were largely self-selected following an open invitation to all team members, and a request for at least one member of the constituent disciplines represented in each clinic. We were therefore able to access the perspectives of physicians, physiotherapists and clinical psychologists comprising the core team of each clinic. Some clinics also included nurse specialists and acupuncturists and these were included in our overall sample.

### Interview process

The interviews were conducted around a topic guide, developed in part from our previous systematic review and study of pain clinic patients [14,15] and constructed to probe respondents for their approach to, and beliefs about chronic pain management. This guide explored practitioners' approaches to managing patients within the pain clinic, expected outcomes following these strategies, practitioners definitions of success, and what they thought patients expected of them.

Interviews were tape recorded and transcribed. Semi-structured interviews limited the opportunity for open ended questions but interviewees were invited to articulate their developed thoughts where appropriate, particularly with regard to managing patients with complex problems. We continued interviews until the emergent themes from the data were sufficiently conceptualised as to not warrant further interviews.

### Data analysis

Textual analysis of the interview transcripts commenced with the construction of a thematic framework to identify key issues and construct concepts. The data were systematically coded, and synthesised by theme [16]. The processes for summarising and coding the data, and arriving at reliable and verified conclusions, conformed to standard conventions of qualitative analysis [17,18]. All transcripts were anonymised; all names used in the results section are aliases assigned for an easier presentation of the data analysis. Regular review and discussion amongst the authors, of evolving themes contributed to the data synthesis and interpretation. Data were coded initially by GH and the coding frame, together with the subthemes, independently scrutinised by SP to determine their robustness. Minor differences of interpretation were resolved by discussion. Particular attention was paid to anomalous data - accounts from one clinic that did not support our theoretical analysis. The study protocol and interview schedule received MREC approval from the South East Multi-Centre Research Ethics Committee (06/MRE01/17).

## Results

Our participants covered the spectrum of staff working in pain clinics (Table 1). Four themes were generated from our data: education for adaptation; re-framing chronic pain as a problem of cognition; managing patients' expectations, and professional boundaries.

**Table 1:** Participant details

Participant's Discipline	Pain Clinical lead	Commitment
Consultant Pain Management Harry	Consultant Pain Management	Full-time

Anaesthetist Alicia	Anaesthetist	Part-time

Anaesthetist Barry	Anaesthetist	Part-time

Anaesthetist Charles	Joint Anaesthetist/Rheumatologist	Part-time

Anaesthetist Derrick	Anaesthetist	Part-time

Anaesthetist Peter	Anaesthetist	Part-time

Anaesthetist Errol	Joint Anaesthetist/Rheumatologist	Part-time

Physiotherapist Andrew	Clinical Psychologist	Part-time

Physiotherapist Betty	Clinical Psychologist	Full time

Physiotherapist Colin	Anaesthetist	Part-time

Physiotherapist/Acupuncturist Doreen	Consultant in Pain Management	Part-time

Physiotherapist Eugene	Joint Anaesthetist/Rheumatologist	Part-time

Clinical Psychologist Archie	Clinical Psychologist	Full-time

Clinical Psychologist Ben	Anaesthetist	Full-time

Clinical Psychologist Callum	Anaesthetist	Full-time

Clinical Psychologist Dawn	Rheumatologist	Part-time

Clinical Psychologist Edith	Rheumatologist	Part-time

Clinical Psychologist Freda	Consultant in Pain Management	Full time

Nurse Specialist Alice	Anaesthetist	Full-time

Nurse Specialist Beatrice	Anaesthetist	Part-time

Nurse Specialist Carol	Consultant in Pain Management	Full-time

Chinese Acupuncturist Adam	Consultant in Pain Management	Full-time

Administrator Adele	Anaesthetist	Full-time

Rheumatologist Aidan	Rheumatologist	Part-time

Rheumatologist Bernard	Joint Anaesthetist/Rheumatologist	Part-time

Analysis of these themes [19] explored the nature of chronic pain management and considered the means by which the pervasive and dominant model of pain management (bio-psychosocial) was interpreted and accepted by the team members as appropriate [20].

### Theme One: Education for adaptation

For patients assessed by specialist clinicians in pain clinics, failure to identify an underlying physical cause and exclusion of the potential for biomedical treatment impacts on the relationship between the clinician, the patient, and their pain. In such cases the pursuit of a 'cure' or even the possibility of a marked reduction in pain may be discounted by clinicians. While some practitioners report that patients engage with the notion that a cure is not possible, others present more of a challenge for the team to manage. For these the team's task was to shift patients' attention away from relieving or reducing pain by biomedical interventions, towards living with and adapting to pain.

*one of the things we try to ...err... help patients to understand is that this is a different kind of treatment insofar as we're giving them skills to help themselves and it's no longer a medical context in which things are done to them...we do try to empower patients to learn skills which will enable them to be in control of their chronic condition...We tell people we're dealing with the problems the pain causes, rather than the pain itself*. Psychologist (Archie)

The consultation's focus moves from the pain itself as the problem, towards the patients' responses to the pain. For clinicians the imperative becomes one of moving the patient away from passive dependency on biomedicine for a cure or marked reduction of their pain, towards active dependency on the pain clinic as a source of the skills and abilities necessary to enable the patient, upon discharge or completion of their programme of therapy, to cope with their pain and not continue to seek further consultations.

*...we're not working on a medical model, trying to cure people, we're teaching people how to manage their pain and return to a normal lifestyle. So many people come here thinking it's the last resort and they've got nothing to lose really... and they start the education process that they'll suddenly turn round and say, ... this should be happening early on*. Physiotherapist (Andrew)

The clinic team therefore serves an educational function for patients - the objective being to adjust the patient's model of pain from one in which there is a medical 'cure', to one in which the patient is educated to accept that the pain will not necessarily be cured and that ability to manage this pain has a psychological element. This model centres on arriving at a concordance of understanding of chronic pain. This involves setting a clear agenda for chronic pain patients in which the notion of a medical cure for their pain is, in the context of the pain clinic, inappropriate. Rather, the pain clinic team's objectives are to facilitate patients to take responsibility for their own pain management. This requires the team to work with a model of pain that acknowledges the biomedical basis of chronic pain but is not evoked in the consultations with the patient. Instead a psychological model of pain is the central focus of the consultation, with psychosocial interventions such as acceptance, planning, support, limits, and learning to pace physical activities playing an important role.

Instruction into the skills necessary to cope with and adapt to their chronic pain, then, becomes a central part of the bio-psychosocial approach. However, it was widely reported that some patients want to get rid of their pain - not confront it. The clinic staff therefore have to work towards getting patients 'on side', which invariably translates as preparing the patient to take responsibility for their pain - assuming other than a paternalistic, dependent bio-medically based relationship.

*I think education, reversal of physical de-conditioning and improvement of quality of life and coping skills are the important things. I'm not particularly concerned whether I decrease their pain score or not and I would hope by the end of the episode, the treatment episode, the patient feels the same way. So it's really sharing a shared model of what their pain is, and what's important to them is very important, so if they don't see it the same way that you do you're not going to get very far*. Anaesthetist (Barry)

It was evident that most, if not all team members, assumed patients come to the clinic with a sense of dependency which the staff had then to wean them off. This assumes also that they would not wish to be involved in their pain management but have to be encouraged to do so, and further, that patients need to be convinced of an alternative to the biomedical approach. The objectives of the clinic then are to try and encourage patients to understand their chronic pain as an entity which cannot be cured by biomedicine and to foster a new perspective on their chronic pain.

The challenge for the team is not simply to educate patients in a set of skills and coping techniques for patients who otherwise expect a cure or a reduction in their pain, but to enable patients themselves to work towards changing their perspective on their pain.

*...it's not just about giving people strategies, it's about helping people through that process whereby they can accept that pain will happen to all of us and in some cases pain will remain with some people and that it isn't that they're being singled out, I think it is more of a philosophical stance perhaps that we're trying to help people explore*. Psychologist (Dawn)

For the patient, exploring this philosophical stance may involve a lengthy process of coming to terms with chronic pain and accepting the limitations of biomedicine. Within the time constraints of the clinic, devising means of engaging patients with this process at the outset involved a variety of strategies most often instigated by the lead clinician *...what we're trying to do is give them a compelling story that suggests to them they shouldn't even try the physical stuff*. Pain Management Consultant (Harry)

### Theme two: Re-framing chronic pain as a problem of cognition

Patients presenting to pain clinics may have already been screened for any obvious underlying physiological causes by the referring medical practitioner and/or allied health professional. The initial assessment of such patients in most pain clinics was undertaken by the lead clinician (initial assessment was shared with specialist nurse in two clinics) - invariably a consultant anaesthetist whose role included diagnosing conditions requiring medical interventions and where appropriate distinguishing these from cases assessed to require rehabilitation.

Where a purely interventionist biomedical approach (injections etc) was deemed inappropriate, management of patients with chronic pain was directed by a holistic bio-psychosocial model.

*Every patient I see has a full bio-psychosocial assessment by me, so not by a physiotherapist and not by a psychologist, but I'm experienced in assessing patients that way and I'm perfectly happy to consider the patients' problem to be largely psychosocial, psychological, or social, or biological, depending on what the problem is. So I don't have any weighting in my assessment*. Anaesthetist (Barry)

A key element of this model is its emphasis on the patients' cognitions concerning their pain. This process of re-framing a condition from something that can be resolved or markedly reduced by medical intervention, to that of a more complex condition requiring a multidisciplinary approach to assist the person in learning to cope with it requires pain clinic practitioners to carefully manage patients who may otherwise interpret this reframing as tantamount to being abandoned by biomedicine with an associated loss of hope for improvement.

Undertaking a bio-psychosocial assessment and sanctioning this multidisciplinary treatment model reflects the complex nature of managing chronic pain for which there is no attributable, underlying physical cause. It was evident from pain clinic practitioners we interviewed, that while they espoused a holistic approach - combining multiple disciplinary approaches rather than an incremental approach i.e. *there's nothing more I can do, I'm sending you to our physio*, in practice patients' chronic pain was re-framed as a cognitive problem. Specialist lead clinicians trained in interventionist medicine, whilst aware of the boundaries of their knowledge of cognitive based treatment, may have limited expertise in assessing cognitive conditions, compared with clinical psychologists.

*I think I can do a basic psychosocial assessment and determine whether that's an important component of the problem, but I wouldn't feel comfortable taking it any further than that*.... Anaesthetist (Barry)

Moreover their role in the clinic for many is part time and distinct from their other medical diagnostic role. Others regard their experience in managing chronic pain as equipping them to cross boundaries and assume a dual role - as both diagnostic clinician or practitioner and cognitive-based therapist.

*I may be leaning more towards the psychological approach as well, whereas I do perform injections and I do all that, and I do quite a lot of injections and tablets etc, etc. I also work every other week with psychologists in a combined clinic, so my way of looking at things, I think, is very much determined by my ongoing experience there. .. So I look at a bio-psychosocial assessment as the crux of assessing any patient*. Anaesthetist (Alicia)

*...sometimes I think of myself as a psycho-physiotherapist, I'm very clear that I'm not a psychologist, so when somebody presents with something difficult I just say, okay, that's very interesting, but I can't really talk to you about that, don't really know where to go with that. But if they say I just can't get round to doing anything, then I feel that you need to be a psycho-physiotherapist to get round that, to do with motivation, as I have in that area quite happily...I think we tend to be tellers - stand up, sit down, lift your leg up - and I like being an asker, I think I've learnt to be an asker*. Physiotherapist (Eugene)

Assessing patient need as requiring either intervention or self-management (rehabilitation) or both is therefore determined by practitioners whose primary expertise is interventionist (bio-medical) and who assess patients with little or no input from other members of the team. Thus there was some acknowledgement that part-time pain clinic clinicians were perhaps not necessarily best placed to assess chronic pain patients' holistic bio-psychosocial status.

*I think fairly few skills I've got from anaesthetics are in any way appropriate to pain management - sticking needles in is probably about it and that's a very small part of my practice...I think that's one of the big faults of pain management in this country is that we do it part-time*. Anaesthetist (Errol)

Indeed a number of investigators [21,22] recommend a dual-diagnostic system: a biomedical diagnosis and a psychosocial diagnosis. Nonetheless other members of the team subscribed to a cognitive-based model of pain management [23], though this model was one that was not core to their professional identity - and was interpreted as simply involving them in allowing the patient to talk of their chronic pain experience. Consequently physiotherapists, for example, treated chronic pain patients not just by applying manual therapies but also introducing elements of psychological-based therapeutic techniques.

*I think very often you have just a little bit more time with the patient to listen to their side of the story and talk round the subject, rather than indoctrinating them and telling them to go away and what to do, you listen more to what the patient's fears and problems are to help them overcome them themselves really*. Physiotherapist/Acupuncturist(Doreen)

*I think there are lots of psychological stuff and behavioural stuff that you can do with people that will make their lives better. And you sit down from them and you try and figure out what the story is - and the story is often very complicated and often I've got no idea where it's going to start with. But you look for the weaknesses in their story or the problems that they're having, and they're often very psychological*. Physiotherapist (Eugene)

In these instances chronic musculoskeletal pain is framed as a problem of the patients' cognitions, expectations and beliefs. Physiotherapists, nurses and others we interviewed processed patients by drawing on psychological models - and "narrative reasoning" - approaches more closely identified with specialist clinical psychologists [24]. Although consideration of the constituent elements of the bio-psychosocial model would not necessarily feature equally in the management of patients for whom physical and pharmacological interventions were ineffective or inappropriate, consideration of social factors were seldom articulated by multidisciplinary team members.

### Theme three: Managing patients' expectations

A patient perceiving the clinic as the 'end of the treatment road' has implications for their management, particularly when no biomedical intervention is appropriate. Managing the care of such patients is challenging as the patient's expectations may differ from those of the multidisciplinary team. Having a shared agenda which recognises the inappropriateness of biomedical interventions and favouring instead a psychosocial approach, remains the biggest challenge, for practitioners and patients.

*The first thing is to identify with the patient what they want ... err ... which may sound silly, because they attend the pain clinic to get rid of pain. ...And once you've established that they've got chronic pain, you then take them through the journey of symptom management, so you look at what's been tried in the past, what may be worth trying in the future, so we don't abandon all hope straight away. And at the same time we're also looking at the longer term pain management strategies, so getting them to pace themselves, learning to manage pain better, be more in control of it, how it affects them and relationships and social life and work and all these things*. Anaesthetist (Derrick)

This was especially so for some physicians whose stated aims for these patients were to relieve or reduce their pain. When this is not realistic it became necessary to shift patients' expectations towards a focus on coming to terms with their pain by developing coping strategies. This shift of focus away from biomedical interventions was considered by team members to be interpreted by some patients, fearful of being discharged, as indicative of being abandoned, and brought into sharp relief potential differences amongst multidisciplinary teams. Some clinics responded with a policy of not discharging patients but rather maintaining an open-ended relationship based on ongoing support for self-management and independence. Others were blunter with those patients with persistent unrealistic expectations:

*I'll be as blunt as I need to be, I'll say, look, we've done everything we can to try and manage symptoms, we're not going to win. And you present them with the bio psychosocial model, where you say, right, the bio bit doesn't work, so we can look at the psycho bit and the social bit, if you don't want to buy into that then we can't offer anything*. Anaesthetist (David)

..*occasionally you do have to poke them in the eye with, I'm sorry, this is where I think it should stop*. Rheumatologist (Bernard)

This blunt approach was particularly characteristic of some clinicians who triaged such patients as not suitable for intervention, and anticipated patients disappointment at what might be considered abandonment by bio-medicine.

Central to all clinics' management strategies was validating patients' sense of hope - hope for help with their pain - not necessarily reducing it but developing rehabilitation strategies to improve their quality of life. This necessitated establishing patient expectations - some sought only a diagnostic label for their pain. An integral feature of managing patient expectations involved bringing the patients relationship with the clinic to a conclusion. Sometimes managing patients' expectations was more akin to a take it or leave it approach - which potentially serves to exacerbate a sense of abandonment. In this vein coming to terms with chronic pain was considered analogous to the process of bereavement.

*I think the most difficult patients are the ones where there's a disparity between the diagnostic and the management process, i.e. patients who after years of having had the chronic condition still seek a diagnostic confirmation of the problem and still seek out clinicians, often other clinicians, who still support an ongoing diagnostic process. Because a so-called successful voyage into dealing with chronic pain in my perception is that there needs to be a process of acceptance and a process of bereavement*... Anaesthetist (Alicia)

This analogy is particularly appropriate in that it acknowledges the transition from seeking relief or reduction of pain to coping with pain as a complex and often lengthy process - one which an interventionist biomedical model is inappropriate.

### Theme four: Professional boundaries

While boundaries between health workers have undergone marked changes in recent years, the impact of these changes for the specialist pain clinic team is not known. A key feature of pain clinics is the multidisciplinary nature of their pain management programmes with all clinics applying a holistic, bio-psychosocial model of pain management in a professed non-hierarchical structure.

*I think we're all equal and different*. Anaesthetist (Charles)

*I'll bring out the physical and physiotherapy aspects when, say, L (Clinical Psychologist) is doing her psychology bit. And she'll back up and if I'm doing the physical bit she'll say can you see how this influences the psychology? So we back each other up, there's one message going out*. Physiotherapist (Betty)

Multidisciplinary working potentially enables clinics to provide a level of patient benefit that collectively is greater than the sum of the individual team members' contributions. However, this style of working could also undermine the efficiency of the clinic's work. This stems from people working outside the area of their core expertise for which they are trained. Thus although the regulatory body for UK physiotherapists [13] endorses the application of a bio-psychosocial model, many practitioners are not formally trained in all the component parts of this model [25-27]. When extending one's activities into areas beyond one's key competencies professional identities may become blurred. This was especially evident among some, though not all physiotherapists, working with patients on pain management programmes by drawing on both biomedical and cognitive treatment models.

Where professional boundaries were retained, efforts were made to ensure patients experienced integrated rather than disparate care. This shared common purpose was especially evident in accounts of team meetings, whose proceedings were purportedly a forum for all members to contribute equally.

*...every second week we do have a multidisciplinary meeting and everybody's given equal say. And certainly you never know what somebody's really thinking, I suppose. But certainly I get the impression that we're all aware that we have equally important roles to play. And the physiotherapists and nurses - and the managers - do attend our multidisciplinary meeting as well. So I do think its egalitarian*. Anaesthetist (Barry)

Respect for boundaries was clearly evident but with close working relations there was, albeit unwittingly, some boundary encroachment with, for example, physiotherapists managing some patients by drawing on behavioural psychological constructs. For lead physicians - particularly those with a psychology degree - whose management of chronic pain patients included a cognitive therapy approach - this was considered less a blurring of their traditional boundaries but more accurately a case of role expansion in that bio psychosocial model was perceived as an addition to their professional repertoire of roles - introducing a holistic as well as a purely biomedical assessment.

*... that's not to suggest that the consultants will give the medical opinions and the physios or other therapists or the nurses might give the psychological side, in fact quite the contrary, a couple of our consultants here have also got a degree in psychology, so they're obviously coming from that flavour as well. So it's for everybody just to chip in on what they think*. Physiotherapist (Colin)

Team members tended not to encroach on other's territory when initially assessing patients. Rather this assessment, although ostensibly a bio-psychosocial assessment was invariably undertaken by team members whose initial emphasis was on biomedical management followed in turn by psychosocial considerations.

*...at assessment we think about all the 3 aspects and we are always thinking about them integrating. I think in reality - this is just my assumption - I think in reality, yes, they hit the biological first. But then why not, because that's their role - as a psychologist I would maybe get the psychology first*. Psychologist (Ben)

*I work alongside the consultants and basically it's a mostly biomedical assessment in order really to get a reasonable diagnosis or to investigate further*. Physiotherapist (Andrew)

*...the medics initially are after a pain cure, the psychologists aren't, we're after helping people to manage their pain, reduce their distress and live with life despite pain. I think once the medics have gone through their biological bit, can we help them with medication, injections, whatever, I think at that point we're then more all on board together about, okay, it's now time to manage it*. Psychologist (Dawn)

Notwithstanding issues regarding the cohesiveness of the multidisciplinary team in managing patients with chronic pain, all were united in sharing a clear sense of their role being misunderstood by referral agencies.

*..for the vast majority of people who refer here this is considered to be where you go when you can't think of anything else to do*. Anaesthetist (Alicia)

*You also see a lot of patients who are not adequately investigated before they come here, the important point being we should not be a diagnostic clinic, we're a symptom control clinic, we're a pain management clinic *Anaesthetist (Barry)

## Discussion

The application of the bio-psychosocial model for chronic musculoskeletal pain management reflects the complex, multi-levelled nature of chronic musculoskeletal pain. There was an impressive consistency in rhetoric from all team members espousing an integrated bio-psychosocial approach to chronic pain management, though some doctors had problems integrating the physical and psycho-social approaches with the traditional bio-medical model. There was less consistency in opinions about how this should be applied and the psychological elements of the approach predominated. The challenge for the team is to move from doing something *to *patients and instead facilitating patients to adopt behavioural and psychological changes enabling them to self-manage their pain. In addressing this challenge we found the multidisciplinary team's management strategies focused on the psychological domain of the bio-psychosocial model. Whilst this aspect of patients' management might logically belong to specialist clinical psychologists, the ethos of pain clinics as multidisciplinary leads its members (irrespective of lack of appropriate training or otherwise in psychology) to embrace psychological constructs as part of their patients' management plans - a position endorsed by some professional ruling bodies.

However, the model is open to various interpretations. For most, there was a sense that because the patient had been assessed as not appropriate for interventions - by default the management strategy was to focus on the cognitive and behavioural factors - working to rehabilitate patients by altering their expectations and help them develops effective coping strategies. As a result, team members whose core discipline did not encompass cognitive and behavioural elements, such as physiotherapists, physicians and nurses, alongside professionally qualified clinical psychologist experts in cognitive behavioural strategies incorporated these into their management plans.

For some practitioners, managing patients using the bio-psychosocial model leads them to downplay their biomedical disciplinary background in favour of dealing with the patient's chronic pain by addressing their beliefs, expectations and behaviours. Key to this is an assumption that people with chronic pain for which there is no biomedical intervention suitable for their chronic pain would benefit from being "taught" a set of cognitive-based skills as an integral feature of their rehabilitation programme. Consequently the bio-psychosocial model is adopted only partially with most attention given over to cognitive and behavioural factors - coping strategies, and commensurately less to social factors such as the nature, character and level of social support patients are able to access to assist in managing their chronic pain.

Notably absent from the accounts was any acknowledgement or detailed account of how practitioners managed the social aspects of chronic pain i.e. addressing issues relating to how patients social interactions was affected by their chronic pain. For example, issues relating to social support, sense of identity and stigma associated with being a chronic pain sufferer were revealed in our earlier study of patients' perspectives, but these were not broached in any detail among the practitioners interviewed. This contrasts with detailed accounts from a range of practitioners reporting how patients' cognitive and psychological therapeutic needs were managed. It is plausible that implicit in these accounts the patients' social needs were also considered by practitioners. However this was not elaborated upon and there were no accounts framing patients' chronic pain management as requiring consideration of both the patients' cognitive and social adaptive strategies. Whilst this is a feature of many structured pain management programmes which invite participation by patients' relatives (including the pain management unit sampled), it was not evident in the accounts from pain clinic practitioners.

Partial adoption of the bio-psychosocial model in which the pain clinic team members manage chronic pain primarily as a cognitive/behavioural issue as the accepted interpretation of this model is reminiscent of a form of medical hegemony i.e. "*a form of influence based not on coercion but rather on "the normal and easy 'way things go" in the organisation" *[28] in that practitioners reproduce an incomplete multidisciplinary pain management model.

Many of the social aspects of chronic pain, such as housing or financial issues cannot be managed within a hospital clinic as they do not lie within the remit of healthcare professionals in the UK. Even so, clinics were all committed to a bio-psychosocial management strategy promoted by lead clinicians and which serves to justify the application of a multidisciplinary approach in which the disciplinary boundaries of the team become less clearly defined. While this enables a unified team approach to chronic pain management, importantly, variations in its interpretation mean that social factors impinging on patient's ability to manage their chronic pain may be given less consideration in a management pathway emphasising cognitive processes.

It is possible that among the practitioners we interviewed, they assessed a sole psychological approach to be most appropriate for patients for whom interventions were inappropriate or ineffective to self manage, having already considered any relevant social components. That they did not articulate which modalities of the bio-psychosocial model they discounted in arriving at a management strategy does not indicate a propensity towards only a cognitive based approach. However, it was the approach utilised by all members of the multidisciplinary team. This would suggest consideration of social factors impacting on chronic pain management strategies were assessed as not as significant and/or relevant to the teams' therapeutic approach. Alternatively it could represent a default approach to which all team members subscribe. If so, this might diminish the potential of a multidisciplinary model for managing patients whose chronic pain is not responsive to interventions or is not considered appropriate.

## Conclusion

Although this is the largest qualitative study to look at the beliefs of staff who manage patients with this problem across a range of clinics and disciplines, our findings may not reflect processes of all pain clinics and professionals both within the UK and in other countries.

Nonetheless the subjects of this study consistently espoused a bio-psychosocial approach to patients with chronic musculoskeletal pain for whom medical interventions were inappropriate. Educating patients to move from expectating a cure for pain and towards learning to live with pain were important goals of the team's work. This concurs well with the views of patients identified in our previous study [14]. We have identified important differences in the degree of integration of biomedical and psycho-social explanatory models of chronic pain. Notably pain clinic practitioners we interviewed readily embraced cognitive/behavioural based management strategies but reported relatively little if any consideration of the impact social factors played in managing chronic pain for patients failing to respond to interventions or for whom interventions were deemed appropriate. Until these aspects are comprehensively embraced and fully integrated alongside the bio-medical and psychological approaches in the management plan, it will remain difficult to meet the challenge of chronic pain.

## Competing interests

The authors declare that they have no competing interests.

## Authors' contributions

GH and MU conceived the study and GH led on its design. GH collected and undertook the initial analysis of the data, drafted the manuscript and its subsequent revisions. MU, AR SP and JC participated in the study's design and contributed to revising the manuscript. SP contributed to validating the analysis. All authors read and approved the final manuscript.

## Pre-publication history

The pre-publication history for this paper can be accessed here:

http://www.biomedcentral.com/1471-2474/11/51/prepub
